# Society Notes

**Published:** 1893-03

**Authors:** T. J. Bennett, R. P. Talley

**Affiliations:** Secretary; President


### Society Notes.

The Twenty-second Quarterly Meeting of the Austin District Medical Society will be held in Austin, Texas, Thursday, March 23, 1893. You are invited to be present and participate in the following programme:

1. "The Relation of the Dental to the Medical Profession," by Dr. J. P. Stansell; discussion opened by Dr. R. P. Talley and Dr. T. J. Tyner.

2. "A Case of Placenta Previa," by Dr. W. T. Richmond; discussion opened by Dr. Sam Cunningham and Dr. Fannie Leake.

3. "Can Nerve Action be accounted for by McLaughlin's 'Physical Theory?' by Dr. F. S. White; discussion opened by Dr. A. N. Denton and Dr. J. W. McLaughlin.

4. "Membranous Laryngitis," by Dr. C. O. Weller; discussion opened by Dr. A. Garwood and Dr. Matt M. Smith.

5. "A Teatment for Fistula in Ano without Cutting Operation," by Dr. T. J. Bennett; discussion opened by Dr. T. D. Wooten and Dr. J. C. Anderson.

6. Voluntary Papers.

7. Reports of Cases.

T. J. BENNETT, Secretary. R. P. TALLEY, President.

**American Electro-Therapeutic Association**—.At the Second Annual Meeting of the American Electro-Therapeutic Association the following officers were elected for the ensuing year:

President, Dr. Augustin H. Goeler, No. 331 West 57th street, New York; First Vice-President, Dr. Wm. F. Hutchinson, Providence, R. I.; Second Vice-President, Dr. W. J. Herdman, Ann Aarbor, Mich.; Secretary, Dr. Margaret A. Cleaves, 66 Madison avenue. New York; Treasurer, R. J. Nunn, 119 Yonk street, Savannah, Ga.

The Third Annual Meeting will be held in Chicago on Sep.t 12th, 13th and 14th, 1893. A cordial invitatiou is extended to all members of the profession interested in electro-therapeutics. Arrangements for special rates on railways and hotels are in progress.

